# Incident light adjustable solar cell by periodic nanolens architecture

**DOI:** 10.1038/srep06879

**Published:** 2014-11-05

**Authors:** Ju-Hyung Yun, Eunsongyi Lee, Hyeong-Ho Park, Dong-Wook Kim, Wayne A. Anderson, Joondong Kim, Natalia M. Litchinitser, Jinwei Zeng, Junsin Yi, M. Melvin David Kumar, Jingbo Sun

**Affiliations:** 1 Department of Electrical Engineering, University at Buffalo, State University of New York, Buffalo, New York 14260, USA; 2 Department of Physics, Ewha Womans University, Seoul 120750, Korea; 3 Patterning Process Department, Nano Process Division, Korea Advanced Nano Fab Center, Suwon 443270, Korea; 4 Department of Electrical Engineering, Incheon National University, Incheon 406772, Korea; 5 College of Information and Communication Engineering, Sungkyunkwan University, Suwon 440746, Korea

## Abstract

Could nanostructures act as lenses to focus incident light for efficient utilization of photovoltaics? Is it possible, in order to avoid serious recombination loss, to realize periodic nanostructures in solar cells without direct etching in a light absorbing semiconductor? Here we propose and demonstrate a promising architecture to shape nanolenses on a planar semiconductor. Optically transparent and electrically conductive nanolenses simultaneously provide the optical benefit of modulating the incident light and the electrical advantage of supporting carrier transportation. A transparent indium-tin-oxide (ITO) nanolens was designed to focus the incident light-spectrum in focal lengths overlapping to a strong electric field region for high carrier collection efficiency. The ITO nanolens effectively broadens near-zero reflection and provides high tolerance to the incident light angles. We present a record high light-conversion efficiency of 16.0% for a periodic nanostructured Si solar cell.

Many approaches have been reported to achieve low-cost^1^,^2^,^3^,^4^,^5^,^6^,^7^ and high-performing photovoltaics (PVs) using designs for light-incident surficial structures^3^,^8^,^9^,^10^,^11^,^12^,^13^, and/or optical behaviors^14^,^15^,^16^,^17^,^18^. Recently, great interest has been focused on the periodic structure of light-active materials for optical enhancement^19^,^20^,^21^,^22^. Lambertian light trapping is proposed for an ideally random surface, which can extend the optical path length by 4n^2^ or higher^21^,^23^,^24^, where n is the refractive index. A strong candidate for extraordinary optical enhancement by 14.5n^2^ has been proposed^25^,^26^ using a periodic nanoscale grating structure^27^.

Microscale designs are attractive for fabricating structures using a commercial method for large scale application^11^,^12^,^13^. Nanoscale architectures are definitely promising in attempts to reduce light-reflection with an enormous enlargement of the light-active surface area; however, it is extremely difficult to establish a neutral region^28^,^29^ or a space charge region (SCR)^11^,^13^,^30^ for nanoscale entities due to their tiny geometry. The SCR has the highest carrier collection efficiency, benefitting from a strong electric field (*E*) existing inside the region. Recent investigations have revealed that the position of an SCR is peculiarly crucial inside light-absorbing entities^11^,^13^.

Though the ITO nanowire and nanorod structures have been employed in photovoltaic and LED applications^31^,^32^,^33^, there are limitations in shaping the junctions of the nanoscale devices^9^,^34^. A direct etching method has been typically used to pattern semiconducting material. However, the direct etching of a semiconductor almost always leads to surface defects^3^,^24^,^27^,^28^,^30^,^35^. Such defects in the semiconducting material readily cause low carrier collection efficiency^34^, resulting in degraded solar cell performance of ~10% or less for Si and 13.8% for compound semiconductors; those achievements^5^,^27^,^28^,^30^,^36^ are far behind from the pursued efficiency of over 20% of Si solar cells^6^,^22^,^35^,^37^.

The defect-induced recombination loss is a main reason to cause a discrepancy between the optical benefit and the electrical degradation of the direct nanoscale patterned semiconductor PVs. And thus, an urgent issue is assigned to the nanostructured PVs active light management^23^,^38^. Is it possible to form the optically beneficial nanostructures without electrical recombination loss by surface defects of the direct patterned semiconductor?

One promising scheme can be a periodically nanoscale patterned transparent conductor. In this design approach, it is possible to assign nanostructures for a planar semiconductor without a need of the direct etching in semiconducting material.

In this paper, we propose and demonstrate a periodic nanoscale-patterned high performing Si solar cell, without direct etching in a light-active semiconductor. This design is realized by an imprint method to form nanoscale transparent patterns on a bare 4-inch Si wafer.

In this paper the following challenges are addressed:

-  Fabrication of the periodically nanostructured PV device without a direct etching of the semiconducting material.Optically transparent indium-tin-oxide (ITO) nanolenses were periodically formed on a planar Si, which enables to fabricate the nanostructure PV device without damage to the semiconductor. And thus, the light-absorbing semiconductor (Si) is free from the surface defects, which are readily caused by the direct etching of the semiconductor. This approach ideally provides no etching-induced recombination loss from the semiconductor.

-  Optical and electrical functions of the ITO nanolensThe incident light has a form of plane wave and passes a planar semiconductor without an optical modulation. We have optimized the ITO nanolens to work as an optical lens by tuning the propagation length of the incident light. The nanolens geometries were designed to focus the various photon wavelengths at a designated spot to maximize the collection efficiency of photo-generated carriers. In terms of an electrical aspect, the electrical conducting ITO nanolens-arrays support the photo-generated carrier transport.

-  Reflection minimization for the incident photon wavelengthsTo improve solar cell performances, it is essential to drive the incident light into a light-absorber with little reflection at the surface. The ITO material has an intermediate refractive index value between air and Si. And thus, an ITO layer insertion spontaneously reduces the light reflection. We demonstrated an effective scheme of near-zero reflection for a broad range of wavelengths by using the ITO nanolenses.

-  High tolerance to the incident light anglesNot only is the solar cell efficiency important, but also it is a crucial issue to extend the solar utilization time, which directly increases the solar power generation. Unfortunately, the incident light angle to a solar cell varies with time during the day and throughout the year. This limits the power generation time for a conventional solar cell. The ITO nanolenses-embedded solar cell shows a strong potential of the high tolerance to broad incident light angles.

-  A spatial overlapping of the photo-generated carrier region and the SCR location to enhance the carrier collection efficiency.The incident light generates the photo-generated carriers in the light-absorbing Si material and establishes the light-induced *E*. For a solar cell, there exists a built-in *E* in the SCR, which is a driving force to collect photo-generated carriers. It is reported that few of the nanostructure systems such as metallic nanospheres^39^, silver nanorods^40^ have been used to focus and magnify the broadband color images through localized surface plasmon (LSP) resonance. In the present work, a nanolens system is applied in renewable energy generation. Hence, the nanolens was designed to a focus broadband solar spectrum over the SCR rather than transfer the specific color and magnitude information for imaging. The more important geometric factor of the nanolens is the radius, which determines the focal length of the light. The spatial overlapping of the light-induced *E* and the built-in *E* due to focusing the photon wavelengths over SCR enhances the carrier collection efficiency^41^.

## Results

### Formation of nanolens-arrays

**Figure 1:**
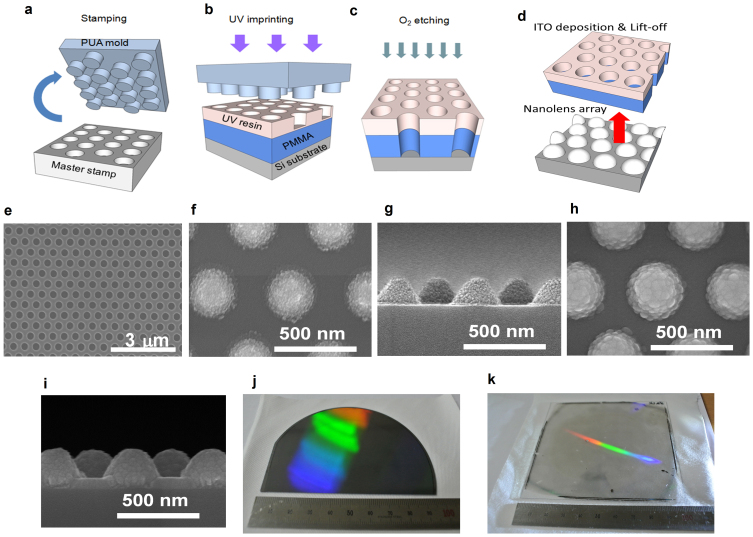
Formation of nanolens-arrays. (a–d), Schematics of nanoimprint method. (e), A scanning electron microscopy (SEM) image of PMMA hole-arrayed patterns. SEM images of ITO-nanolens-arrays (f, g), and ITO-nanolens-arrays with an additional 80 nm-thick ITO film (h, i). Photos of ITO-nanolens-arrays on Si wafer (j) and on glass (k). For the Si wafer, the n-p junction was previously formed using a doping process (See Methods).

Figure 1 shows the schematic of the proposed structure. Nanostructure features were embedded in an optically transparent ITO layer to fabricate nanolenses, instead of using a Si substrate. This approach properly formed the periodically nanostructured PV device without a direct etching process to Si.

In order to fabricate the proposed ITO nanolens structure, we used a nanoimprint method to fabricate nanoscale patterns, which is suitable for patterning of large-scale periodic structures with low fabrication cost and high throughput^42^. Our nanosize hole-array was patterned on a 4-inch Si wafer as a target substrate. A polymer mold (polyurethane acrylate, PUA) was used to inversely replicate the structure from a master stamp (Fig. 1a). The UV imprinting process was performed to pattern hole-arrays on the target Si substrate (Fig. 1b). The inversely replicated PUA mold was then pressed against the UV-curable resin. By detaching the PUA mold from the UV-irradiated resin, the hole-arrays of the imprinted resin layer were produced on a 200 nm-thick polymethyl methacrylate (PMMA) layer. Subsequently, an O_2_-etching process was used to pattern identical hole-arrays on a PMMA layer (Fig. 1c, d, e).

We deposited a 200 nm-thick ITO film on the PMMA hole-array Si substrate. A lift-off process was performed to remove the PMMA layer in an acetone solution under 15 min of sonication; there remained a 200 nm-height ITO-nanolens (with a radius of 180 nm) array on the Si substrate (See Methods), as presented in the top-view image (Fig. 1f) and in the cross-sectional view image (Fig. 1g). Each ITO-nanolens spontaneously contacts to the Si substrate; however, it is electrically isolated from the neighboring nanolenses. An additional an ITO film (80 nm) was coated on the previously formed 200 nm-height ITO-nanolens-arrays on the Si substrate (Fig. 1h). A cross sectional image (Fig. 1i) clearly depicts the ITO-film coating layer on the ITO nanolenses and the Si substrate, which ensures the electrical connection through the entire nanolens-arrays.

As a result, the front surface has nanoscale patterns comprised of an 80 nm-thick ITO film layer with 200 nm-height ITO-nanolens-arrays. Electrical conduction was obtained from this front surface; the device was found to have a low sheet resistance of 29.4 Ω/sq, which will ensure good electrical contact^12^,^43^,^44^. The optical transmittance was measured and found to be 90.95%. Photographic images are presented for the ITO nanolens-array patterned on the 4-inch Si wafer (Fig. 1j) and the glass substrate (Fig. 1k).

### Reflection profiles for the flat ITO film, nanolens-arrays, and nanolens-arrays with ITO film-coating on a Si substrate

**Figure 2:**
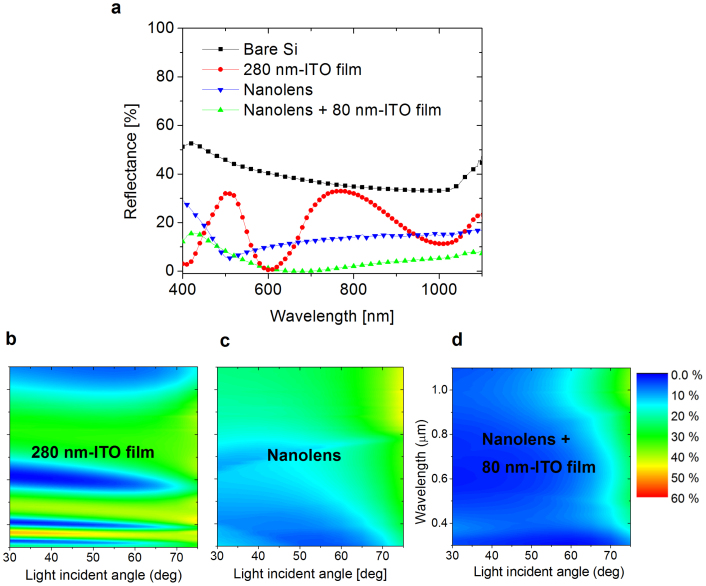
Reflection profiles for the flat ITO film, nanolens-arrays, and nanolens-arrays with ITO film-coating on a Si substrate. (a), Nanolens-array with an additional ITO film coating significantly suppresses the reflection by 4.7% for λ = 400–1100 nm. (b–d), Angle-dependent reflectance profiles as color maps.

In order to characterize the ITO-nanolens structure, we performed the following optical and electrical measurements. A reduction in reflection is crucial to drive more photons into a light-absorber. We measured reflectance profiles from various ITO structures on a Si substrate (Fig. 2a). A flat 280 nm ITO film was prepared to compare the reflection features of a 200 nm-height ITO nanolens-array (Nanolens) and a 200 nm-height ITO-nanolens-array with a coating of 80 nm-thick ITO film (Nanolens + 80 nm-ITO film). The thickness of the additional 80 nm ITO coating film was chosen using an optical interfacial design for bare Si (See Methods), in order to minimize the reflectance^28^,^44^.

Average reflectance was found in the range of 400–1100 nm. A bare Si substrate gave a high reflection value of 38.90%. Meanwhile, a 280 nm-ITO film embedded Si substrate moderately reduced the reflection value to 18.63%. A nanolens-embedded surface more effectively reduced the reflection value (13.85%). A minimum reflection point (5.39%) was found at λ = 510 nm, without touching the zero reflection point.

A further reduction of the reflection of 4.70% was achieved from a nanolens with an ITO film-coating surface. The additional ITO film-coating fully covered the surface without leaving behind a bare Si region. It is worthwhile to note that this structure provides broad near-zero reflection (R < 1%) for wavelengths between 608 nm and 751 nm.

A practical solar cell has a peak electric power generation condition, in which the light incident angle is normal to the solar cell, having a zero angle value. However, the incident angle is time-varying and therefore a sustainably high tolerance of the independence to the incident light angle is an important factor^8^,^10^,^18^,^21^,^45^ to improve the solar cell power-generation amount and the utilization time as well.

An ellipsometric system was used to measure the angle-dependent reflectance profiles for the flat ITO film, nanolens-arrays, and nanolens-arrays with ITO film-coating on a Si substrate with incident angle variations from 30° to 75°; data are presented as color maps (Fig. 2b–d). The flat ITO film has very limited wavelength range for zero-reflection and showed substantial reflectance profiles for short-wavelengths, indicating crucial angle dependencies. Meanwhile, the nanolens surface seems to be efficient at reducing reflectance values for wavelength variations. In addition, this structure sufficiently relieves the incident angle dependency. A further improvement has clearly been shown with the nanolens-arrays with ITO film. This structure significantly expands the near-zero reflection regions with a high tolerance to incident-light angles for broader wavelengths (Supplementary section ‘Weighted- reflectance').

### Light distribution according to wavelengths

The transparent nanolens structure has a convex feature, and thus can work as a lens. In conventional optics, focusing size and depth are limited by diffraction phenomenon^46^,^47^. A solid immersion lens has been shown to have the potential to overcome the diffraction limit by filling an object space with a solid material, that has a high-refractive index^47^,^48^,^49^,^50^.

The ITO-nanolens can provide an active solution for efficient light management; it can focus the incident light into a light-absorber. When a plane wave is incident on the ITO-nanolens, the light is refracted by the interface curvature and establishes a length that can be focused. We have investigated the focal length (F_lens_) formation for short, medium, and long wavelengths, as shown in Table 1 (also see Methods).

**Figure 3:**
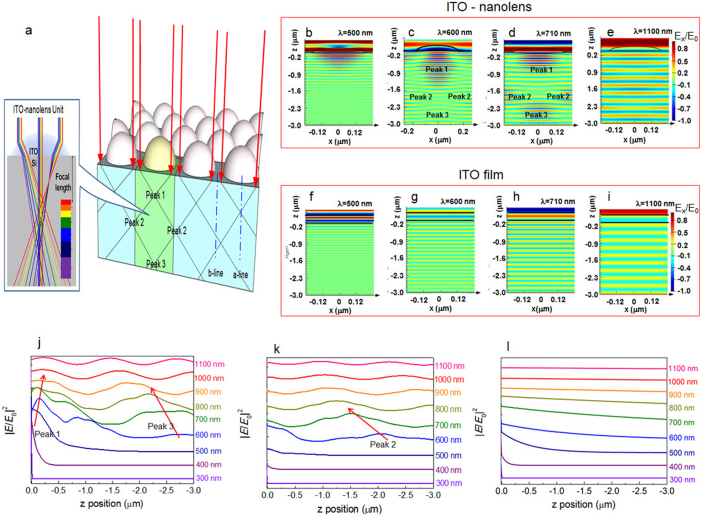
Light distribution according to wavelengths. (a), An ITO-nanolens effectively modulates the visible light into the Si absorber. (b–f), FDTD simulation shows strong electric distribution with ITO-nanolens. (f–i), Incident light moves as plane waves through a flat ITO film. Strong electric peaks were observed along the (j) ‘a-line' and (k) ‘b-line'. (l) The flat ITO film shows only shallow electric fields for short wavelengths.

The behaviors of refraction tuning are directly related to the F_lens_ formation, which is determined according to wavelength variations (Supplementary section ‘Refraction tuning'). In the visible range, the ITO-nanolens effectively modulates the light propagation, focusing the shorter wavelength light into a deeper Si absorber and the longer wavelength light into a thinner Si absorber (Fig. 3a).

The light-induced electric field (*E_light_*) intensity distribution in a Si absorber is controlled by two factors. First, the absorption coefficient of Si (α_si_) is high at short-wavelengths^51^, resulting in fast decay of the field intensity in Si. Second, the F_lens_ of the nanolens is gradually decreased, with increased wavelengths in the visible range (Table 1), due to the reduction of the refractive index of Si (n_Si_).

In order to investigate the optical interaction and the *E_light_* distribution of the ITO-nanolens-embedded Si, a numerical simulation based on a finite-difference time-domain (FDTD) calculation (Supplementary section ‘FDTD simulation') was performed for the ITO-nanolens/Si unit cell according to various wavelength photons (Fig. 3b–e). Comparison results are also presented for the flat-ITO film on the Si substrate (Fig. 3f–i). For the flat ITO film, the field magnitude is readily exponentially decayed in the Si absorber due to the increasing distance from the surface (Fig. 3l). In contrast, the plot of *E_x_*/*E*_0_ of the ITO-nanolens array has characteristic patterns by modulating of the incident light, demonstrating the tendency of a strong *E_light_* to appear along the z-direction of the Si depth (Fig. 3j,k).

The ITO front surface consists of the nanolens-arrays. Thus, the light focused by the nanolens will meet the light from the neighboring nanolenses, which can cause interference. As a result, the lens array will generate second and third peaks (Fig. 3a). The FDTD calculations show the field pattern, consistent with expectations for the intermediate wavelengths. For light wavelengths (*λ*) of 600 nm and 710 nm, the Si absorber holds a moderate value of α_si_. The first focusing peak appears close to the surface of the Si absorber. Neighboring nanolenses adjust the further light behavior, forming the second and third peak generations to the Si depth, (Fig. 3c,d). The second peak generation is distinctively found along the ‘b-line', which is the center-point of neighboring nanolenses in the z-direction (Fig. 3l). The third peak is formed along the ‘a-line', which is a direction of a single nanolens in the z-direction (Fig. 3j). This light redirection phenomenon of incident light is uniquely observed for the ITO-nanolens structure, different from the multiple peak appearances of the tailored Si structure^21^ or the light propagation by scattering^38^.

At a short-wavelength of 500 nm, the *E_light_* intensity distribution shows a dominant first peak, close to an ITO-Si interface (Fig. 3j). No second or third peak can be clearly found at λ = 500 nm due to the higher α_si_ value at short-wavelengths, which results in fast decay of the field intensity (Fig. 3b).

For long-wavelengths (e.g., λ = 1,100 nm), no significant peak can be clearly seen. The low refractive index of Si is the main physical origin that results in the weak lens effect. The incident photons are almost uniformly distributed in the Si absorber through the flat ITO-film without redirection of the incident light^16^. The span of the *E* is directly proportional to the wavelengths of the incident plane waves (Fig. 3f–i). The flat ITO-film surface does not have an effect of focusing the incident light and thus, the light absorption of Si is a function of the distance from the surface, at a fixed wavelength. For a long-wavelength, the period of the peak *E_light_* is extended compared to that of a short-wavelength. This is attributed to the increased span of the *E_light_* distribution according to the increases of wavelength. The long-wavelength photons induce relatively little contribution due to the low α_si_ value for plane wave propagation. Meanwhile, the nanolens seems to be an appropriate solution to improve the utilization of long-wavelength photons for Si solar cells.

The generation rate of photo-carriers is proportional to the field intensity in a Si absorber. Thus, a spatial distribution of the photo-carriers can be readily inferred from the *E_light_* intensity distributions. Practically, some of the photo-generated carriers are recombined, limiting the energy conversion efficiency of the solar cell. The SCR holds a strong built-in potential (0.786 V, Supplementary section ‘Built-in potential'), which is a driving force to sweep the photo-generated minority carriers (electrons in p-Si and holes in n-Si) in opposite directions^11^.

A strong built-in *E* develops inside the SCR (*E_SCR_*) and the probability of photo-generated carrier collection is ideally unity in the SCR. Otherwise, a significant decay of the collection probability occurs in the neutral region (p-Si) with an increasing distance from the SCR due to the exponential decrease of photo-generated electrons. In addition, the carriers should be diffusively collected without assistance of the *E_SCR_*. Meanwhile, a heavily doped emitter layer (n-Si) is an easy spot for serious recombination of photo-generated carriers^11^,^29^. This suggests an important clue. An enhanced performance solar cell may be achieved using a spatial overlapping of the high carrier generation region (*E_light_*) and the space of the high carrier collection probability of SCR (*E_SCR_*).

### Solar cell performances

**Figure 4:**
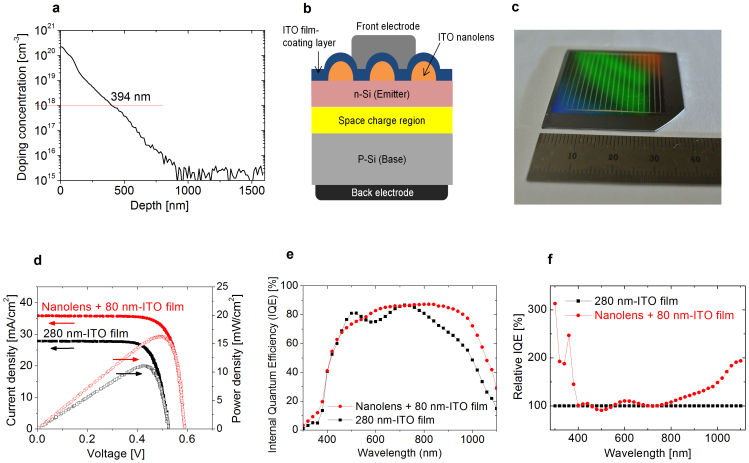
Solar cells: (a), Doping profile for emitter layer (see Methods). (b), Schematic of ITO-nanolens-embedded Si solar cell. (c), Photo of a fabricated solar cell. (d), Solar cell performances under one-sun illumination. (e–f), Comparison in quantum efficiencies for ITO-nanolens device and flat ITO device.

According to the doping profile, we have measured the emitter thickness and found it to be 394 nm (Fig. 4a, see Methods). Considering the heavy dopant concentrations (10^18^/cm^3^ or higher), it is desirable to form a long F_lens_ beyond the emitter thickness to minimize the serious recombination loss at the surface. The SCR width of our cells was 572 nm (Supplementary section ‘SCR analysis'). Therefore, an effective *E* distribution should be positioned at a depth of 394–966 nm, where the SCR is located from the surface. The nanolens feature was designed with this consideration and fully satisfies the focal lengths in order to be positioned in the SCR for broad-band wavelengths (Table 1).

The geometric layout of the ITO-nanolens solar cell is presented as a schematic diagram (Fig. 4b). A relatively large-size nanolens-embedded solar cell was prepared (Fig. 4c) and we characterized light-performance, under one-sun illumination (see Methods). The nanolens device provided substantially improved open circuit voltage (590 mV), short circuit current (35.82 mA/cm^2^), and a conversion efficiency (16.0%) from 520 mV, 27.80 mA/cm^2^ and 10.9% efficiency of a flat ITO-film solar cell (Fig. 4d).

In what follows we discuss the main optical benefits of the ITO-nanolens solar cell performance. Overall, the ITO-nanolens device has higher internal quantum efficiency (IQE) values for the broad wavelengths than did the planar device (Fig. 4e); this situation was different from that of the direct Si-etched structure^12^,^52^. A clear comparison of the two devices according to wavelength can be seen by plotting the relative IQE values of the ITO-nanolens over those of the ITO film (Fig. 4f). The ITO nanolens collimates light into the SCR and also is effective to reduce reflection.

The ITO film has an intrinsic free carrier loss for short and long-wavelengths, without creating electron-hole pairs^53^,^54^, causing low IQE values for the flat ITO film device. Otherwise, the ITO-nanolens provides improved performance according to the manipulation of the incident light for short, long, and visible wavelength light by propagation, concentration, and tuning effect, respectively.

For short wavelengths, serious ITO loss can be relieved by passing the incident light through the nanolens to extend the short wavelength photon propagation into a deeper area of the Si. This significantly relieves the fast decay of the short-wavelength photons at the interface between the air and the ITO medium. Substantially enhanced IQE values were achieved at short-wavelengths. At λ = 300 nm, the IQE value of the ITO-nanolens was improved by 313% compared to that of the planar device. This is a distinctive advantage of the ITO-nanolens in that it can reduce the inherent ITO reflection loss for short-wavelengths^21^. The nanolens is effective in modulating the light propagation at short wavelengths, driving more photons to head towards the Si light-absorber.

For the visible range, the ITO-nanolens device showed generally improved IQE values. A 10% enhanced IQE value was achieved for the ITO-nanolens device compared to that of the planar device at λ = 600 nm, which is the most important incident light for a Si light-absorber^11^. The nanolens effectively modulates the visible wavelengths. Relatively long-wavelengths of visible light (red region, λ = 600–700 nm) have shorter F_lens_ values (830.5–832.2 nm) than that (882.3 nm) of blue light (λ = 500 nm). The nanolens is efficient in tuning the visible-wavelength photons so that they are positioned in the SCR.

For long-wavelengths, the ITO-nanolens device shows prominently improved IQE performance, demonstrating an active scheme for low photon-energy utilization^49^. Light trapping of 800 nm–1100 nm wavelengths is crucial for Si^2^. At λ = 1100 nm, the IQE value of the ITO-nanolens was enhanced by 193.8%. Incident light moves as a plane wave into the Si absorber through the ITO medium. For long-wavelengths, the Si absorber has insufficient absorption of long wave photons. Thus, a planar ITO film device identically bears a critical problem against a long collection length for minority carriers (electrons) in p-type Si. This typically causes an extremely low QE performance for infrared regimes^55^. Meanwhile, the ITO-nanolens efficiently drives long-wavelength photons to be focused near the SCR. This light-adjustable effect spontaneously resolves the low light absorption limit of Si at long- wavelengths and simultaneously establishes a short collection length. Due to an existing strong *E* in the SCR, the photo-generated carriers can be effectively collected, resulting in improved QE values. The nanolens has a distinctive benefit in being able to concentrate the long-wavelengths into Si.

In summary, we propose a promising approach to nanostructure PVs by using a transparent ITO-nanolens without a direct etching of the semiconductor material and demonstrated a record high light-conversion efficiency of 16.0% among the periodic Si solar cells. A periodic structure of an ITO-nanolens was formed using a commercially viable printing method for large-scale devices. The ITO-nanolens provides optical and electrical benefits for nanostructured PVs. Due to the electrical conducting property, the ITO-nanolens supports the photo-generated carrier transport. For the optical aspects, the ITO-nanolens effectively adjusts the focal lengths for various light wavelengths, resulting in the overlapping of *E_light_* and *E_SCR_* to give a higher carrier collection efficiency. In previous work^12^, a microscale ITO lens was used. However, the efficiency improvement was not significant due to the off-positioned focal length location from SCR. Additionally, the ITO-nanolens can broaden near-zero reflection and provide high tolerance to the incident light angles. Ultimately, these benefits may enhance solar power-generation and utilization time. This optically transparent and electrically conductive nanolens architecture would be a promising scheme for the high-efficient nanostructure PVs.

## Methods

### The p-n junction formation

An emitter layer (n-Si) was formed on a Czochralski (CZ) grown 4-inch p-type (100) Si wafer, having a resistivity of 1–10 Ωcm. Phosphoryl chloride (POCl_3_) was used as a doping agent and was flowed into a doping furnace at 800°C for 40 min. The phosphosilicate glass (PSG) formed during the doping step was removed using buffered hydrofluoric acid (10 wt% HF).

### ITO-coating and design

Nanolens-arrays were deployed on a Si substrate. An additional ITO-coating layer (2^nd^ ITO) was deposited to induce an electrical connection through all the nanolens entities. This is also an important detail that is used to control the high reflection of the bare Si region. A nanolens sitting on a Si region is optically affected by the ITO nanolens; however, a region not covered by an ITO nanolens displays the optical behavior of bare Si. Due to the intermediate refractive index of ITO film for air (n = 1) and Si (n = 3.54 ~ 5.57) systems, the insertion of an ITO film (n = 1.76 ~ 2.12) relieves the sudden changes of refractive indexes and therefore effectively diminishes the reflection. The optimum thickness of the 2^nd^ ITO film was determined to be ~80 nm, after considering a quarter wavelength anti-reflection scheme (*d = λ/4n*), in which *n* is the refractive index of ITO (1.85 at λ = 600 nm). ITO depositions were performed in a dc-sputtering system using a 4-inch target composed of In_2_O_2_ containing 10 wt% SnO_2_ at a working pressure of 5 mTorr under Ar/O_2_ (50/1) ambient condition.

### Calculation of focal lengths

The radius (R) of the ITO nanolens curvature can be achieved as in the following equation: 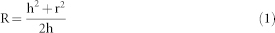
where h is the height of the ITO nanolens (200 nm) and r is the radius (180 nm). Considering these values, we have achieved an R value of 181 nm. In air conditions, the focal length (F_air_) of the ITO nanolens can be found using the following equation: 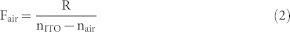
When we consider a Si medium, the focal length is multiplied by the refractive index of Si (n_si_). And thus, the effective focal length of the ITO nanolens (F_lens_) on Si can be extended according to the following sequence^46^: 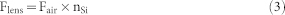


### Emitter layer determination

The emitter depth was measured by secondary ion mass spectroscopy (SIMS, Cameca, magnetic sector ims7f). Regarding the p-Si doping level (~10^16^/cm^3^), a two order higher level (10^18^/cm^3^) was chosen for the n-type doping region in order to give a 394 nm-thickness to the emitter (Fig. 4a).

### Device fabrication and light responses

A flat ITO device and a nanolens device were tailored to a size of 3.2 × 3.2 cm^2^. Solar cell performances were obtained under one-sun illumination using a simulator (McScience, K3000). The profiles of the carrier collection, according to wavelength variations, were obtained using a quantum efficiency measurement system (McScience, K3100).

## Author Contributions

J.K. conceived this research. J.H.Y. designed the ITO-nanolens geometry and fabricated solar cell devices. J.Y. analyzed device performances. H.H.P. performed the nanoimprint method to fabricate the ITO-nanolens-arrays. E.L. performed FDTD simulation. J.Z. and J.S. performed the ellipsometric measurement. D.W.K. supervised E.L. W.A.A. supervised J.-H. N.M.L. supervised J.Z. J.K., D.W.K., W.A.A. and N.M.L. participated in discussion throughout the work and intensively cooperated to analyze results. M.M.D. Kumar analyzed QE performances. All the authors contributed to prepare this manuscript.

## Supplementary Material

Supplementary Information Incident light adjustable solar cell by periodic nanolens architecture

## Figures and Tables

**Table 1:** Focal length profiles for various wavelengths. An ITO-nanolens effectively modulates the incident light to cause long-wavelengths to be shortened and short-wavelengths to be extended

Wavelength (nm)	n_ITO_	F_air_ (nm)	n_Si_	F_lens_ (nm)
400	2.12	161.6	5.57	900.1
500	1.88	205.6	4.29	882.3
600	1.85	212.9	3.9	830.5
710	1.82	220.7	3.77	832.2
1100	1.76	238.1	3.54	843.1
