# Supplementary material for: Incident light adjustable solar cell by periodic nanolens architecture
Source: Sci Rep. 2014 Nov 5;4:6879. doi: 10.1038/srep06879 (PMC4220283; doi:10.1038/srep06879)
Supplement: Supplementary Information — Incident light adjustable solar cell by periodic nanolens architecture [file srep06879-s1.pdf]

Supplementary Information

Incident light adjustable solar cell by periodic nanolens architecture

Ju-Hyung Yun,[1] Eunsongyi Lee,[2] Hyeong-Ho Park,[3] Dong-Wook Kim,[2] Wayne A. Anderson,[1] Joondong Kim,[4]* Natalia M. Litchinitser,[1] Jinwei Zeng[1], Junsin Yi,[5] M. Melvin David Kumar,[4] Jingbo Sun,[1]

[1]Department of Electrical Engineering, University at Buffalo, State University of New York, Buffalo, New York 14260, USA

[2]Department of Physics, Ewha Womans University, Seoul 120750, Korea

[3]Patterning Process Department, Nano Process Division, Korea Advanced Nano Fab Center, Suwon 443270, Korea

[4]Department of Electrical Engineering, Incheon National University, Incheon 406772, Korea

[5]College of Information and Communication Engineering, Sungkyunkwan University, Suwon 440746, Korea

Weighted-reflectance

To estimate the angle-dependent reflection, we have examined the weighted reflectance according to the surface structures. An AM 1.5 G solar spectrum (1000 W/m^2) was used as a light source to measure the reflectance (Supplementary Fig. 1). The weighted reflectance (R_w) was obtained using

$$R_W = \frac{\int_{\lambda=400\,nm}^{\lambda=1100\,nm} R(\lambda)\Phi(\lambda)S(\lambda)d\lambda}{\int_{\lambda=400\,nm}^{\lambda=1100\,nm} \Phi(\lambda)S(\lambda)d\lambda} \qquad (1)$$

where R(λ), Φ(λ) ,and S(λ) are the reflectance of monochromatic light, the incident photon flux, and the internal quantum efficiency, respectively. For the incident angles between 30° and 60°, a 280 nm-thick ITO film showed high R_w values of above 20 %.

Meanwhile, the nanolens-arrayed surface effectively reduced the R_w values. A significantly suppressed R_w was established from the nanolens with an ITO film surface; this can be attributed to the ITO film covering the entire Si substrate. The nanolens with the ITO film surface shows ultralow R_w values (< 5%) below 45° of incident angle.

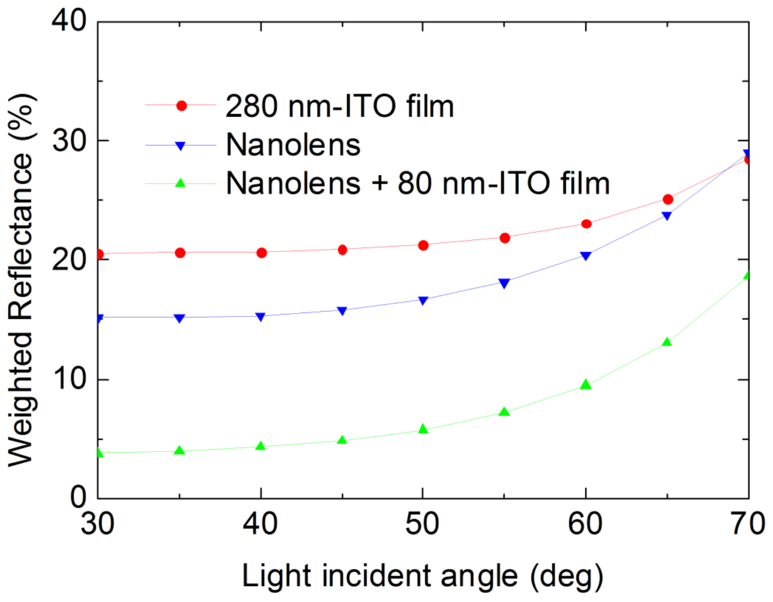

Supplementary Fig. 1 | Angle-dependent weighted-reflectance.

Refraction tuning behaviors of the ITO nanolens

It is useful to understand the light behavior at the ITO-Si interface and the light absorption of the Si material. We can calculate the wavelength-dependent profiles of the incident light propagation[3] through the ITO-nanolens into the Si absorber by using Snell's law ($n_1 \sin\theta_{in} = n_2 \sin\theta_{out}$).

We used a fixed incident angle (θ_{in}) of 30° to the ITO medium (Supplementary Fig. 2) to determine the light behavior for various wavelengths. The refraction angle (θ_{out}) through the Si medium was varied according to the incident wavelengths. A refraction angle of 12.6° was formed at a wavelength of 500 nm; This value is smaller than the value of 14° at 710 nm or the value of 14.4° at 1100 nm.

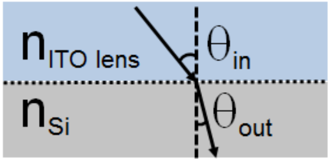

	λ=500nm	λ=710nm	λ=1100nm
$n_{ITO\ lens}$	1.88	1.82	1.76
n_{Si}	4.3	3.77	3.55
θ_{in}/θ_{out}	30°/12.6°	30°/14°	30°/14.4°

Supplementary Fig. 2 | Refraction tuning behavior of the incident angles through the ITO-nanolens to Si.

FDTD simulation

The FDTD method (Lumerical FDTD Solutions) was applied to describe the electromagnetic field distributions of the ITO-nanolens embedded Si system. The flat ITO film-device was also investigated for comparison. A unit cell was modeled by using the periodic boundary conditions. Perfectly matched layer boundary conditions were taken for a unit cell, which completely absorbed the incident radiation. A broad-band pulse (λ= 400-1100 nm) was used to simulate a plane wave incident from the top; the polarization and propagation directions of this wave were parallel to the x-axis and the z-axis, respectively (Supplementary Fig. 3).

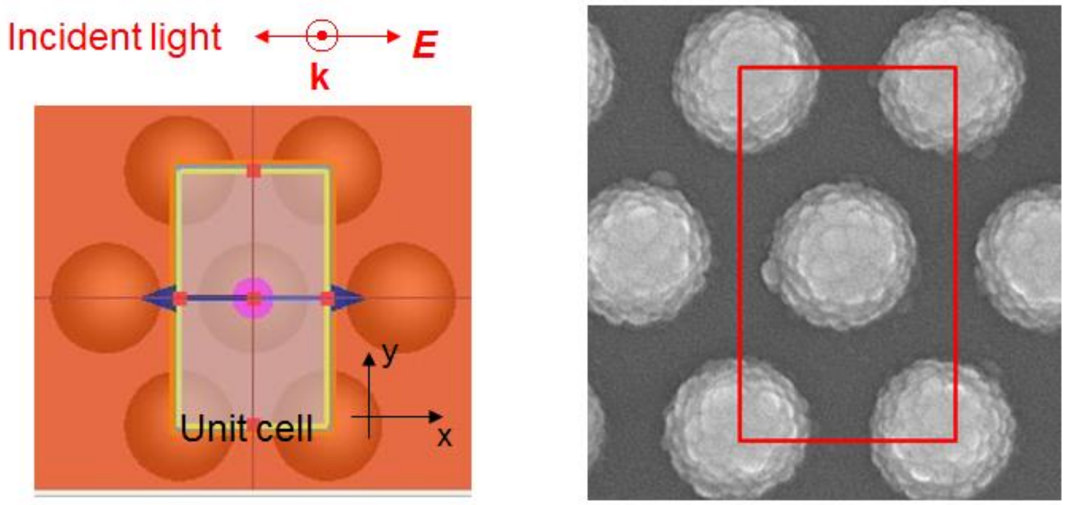

Supplementary Fig. 3 | Schematic illustration of ITO nanolens-arrays used in FDTD simulation.

Analysis of quantum efficiencies

As discussed in the Fig. 4 in the manuscript, the ITO nanolens array collimates light into the SCR, improving internal quantum efficiency (IQE). Moreover, the ITO nanostructure decreases light reflection over all available wavelengths, minimizing incident angle dependency. The combination of lensing effect and minimized reflection result in much improved performance, as presented in the external quantum efficiency (EQE) curve (Supplementary Fig. 4).

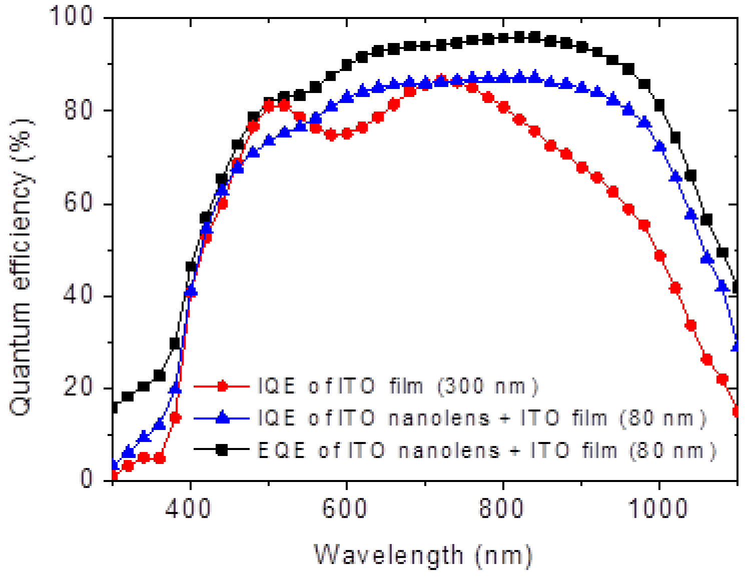

Supplementary Fig. 4 | Comparison of Internal and external quantum efficiency (IQE & EQE) of ITO film Si solar cell and ITO nanolens + ITO film (80 nm) Si solar cell.

Relative contributions of lensing effect and low reflection were qualitatively compared. Contribution from the lensing effect only can be calculated by comparing the IQE of planar ITO and nanolens ITO structured solar cells. Contribution of both lensing effect and low reflection was calculated by EQE data of the ITO nanolens solar cell as seen in Supplementary Fig. 5.

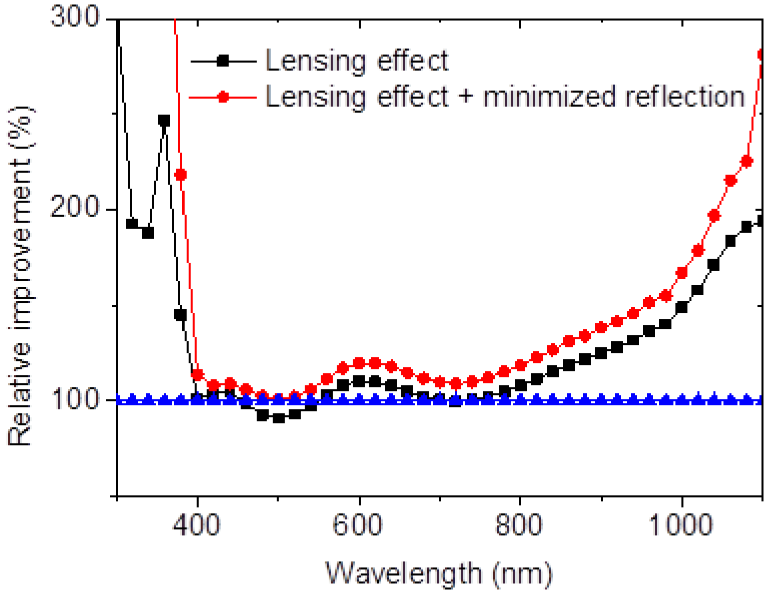

Supplementary Fig. 5| Contribution of the improvement: lensing effect only and lensing effect with low reflection of ITO nanolens solar cell.

Built-in potential

For p-n junction formation, a built-in potential (V_b) is spontaneously established without moving the carriers. Doping levels of the emitter and the base determine the V_b value, according to the following equation:

$$V_b = (kT/e)\ln(N_a N_d/n_i^2) \qquad (2)$$

where k, T, e, N_a, N_d, and n_i are the Boltzman constant, electron charge, temperature, base-doping density, emitter-doping density, and intrinsic carrier density, respectively. By calculation, we extracted the value of V_b as 0.786 V.

SCR analysis

An SCR is spontaneously formed between two different neutral regions after the migration of the free electrons and depleted carriers in the p and n regions. The total SCR width (W) is the sum of W_p into the p-side and W_n into the n-side. Due to the two orders higher doping concentration of the n-side ($10^{18}/cm^3$) compared to that of the p-side ($10^{16}/cm^3$), the most depleted region (99 %) deploys into the p-Si base. An SCR width of 572 nm was obtained by using the following equation.

$$W = (2\varepsilon_0\varepsilon_r V_b/qN_a)^{1/2} \quad (3)$$

where ε_0, ε_r, $V_{b,}$ q, and N_a are the permittivity of vacuum, dielectric constant, built-in potential, quantity of electric charge, and acceptor doping density, respectively.
